# Optimizing control efficiency in discrete-time multi-agent systems via event-triggered containment techniques combining disturbance handling and input delay management

**DOI:** 10.1016/j.heliyon.2024.e33975

**Published:** 2024-07-05

**Authors:** Hanen Louati, Azmat Ullah Khan Niazi, Mhassen. E.E. Dalam, Waqar Ul Hassan, Khawer Hameed Khan, Mohammed Alhagyan

**Affiliations:** a Mathematics Department, Faculty of Science, Northern Border University, Arar, KSA, Saudi Arabia; b Department of Mathematics and Statistics, The University of Lahore, Sargodha 40100, Pakistan; c Department of Mathematics, College of Sciences and Arts (Muhyil), King Khalid University, Muhyil 61421, Saudi Arabia; d Mathematics department, College of Humanities and Science in Al Aflaj, Prince Sattam Bin Abdulaziz University, 11912, Saudi Arabia

**Keywords:** Multi-agent systems, Event-triggered control, Containment control, Disturbance handling, Input delay management

## Abstract

The goal of this paper is to mitigate disturbances and input delays while optimizing controller actuation updates for discrete-time multi-agent systems through the use of an event-triggered confinement control system, especially in resource-constrained scenarios. This approach when combined with event-triggered control techniques, then every follower in the system adjusts its condition at specified times based on an event-triggered condition that is suggested. The containment control system issue in the presence of disturbances and input delays was tackled by using both decentralized and centralized event-triggered control systems. Using matrix theory and the Lyapunov technique, convergence analysis is conducted to show that the proposed strategy stays free of zeno phenomena. Numerical boosts are used to further illustrate the impact of theoretical results.

## 1 Introduction

Cooperative control of multi-agent systems has drawn a lot of interest lately because of its various uses in a variety of domains are sensor networks, cooperative control in mobile robots, and formation of control unmanned vehicles, spacecraft alignment, and so forth [1]. In certain real-world scenarios, multi-agent systems may have more than one leader. In these cases, the main objective is to create a suitable control to protocols that direct the followers into a specific geometric area that the leaders have defined. This is known as the confinement control problem. Applications for containment management in both military and civilian settings appear very promising. For example, when a group of autonomous cars travels from one goal to another, only some of them have the sensors needed to identify potentially dangerous impediments. It is possible to designate certain vehicles as leaders and the other vehicles as followers among the sensor-equipped vehicles. Once the leaders have constructed a moving safety area, the group of vehicles can safely arrive at the destination provided by the followers. A set of some single, double, and higher-order dynamics have been examined in relation to a containment that control the problem [2]. Agents use in multi-agent systems of the future might be outfitted with tiny embedded microprocessors to gather data from nearby agents and take control of actuation in accordance with pre-established rules. As a result, controllers are used on digital platforms, and control laws are only updated seldom. Sampled-data control systems and event-triggered control systems are typically used to address this situation. The agents update their controllers synchronously and periodically in accordance with a constant sampling interval for sampled data control. Note that the sampled-data control is not appropriate for larger-scale networks when energy processing and communication restrictions are explicitly addressed and that it may be conservative given that the constant sampling time must ensure stability in the worst-case scenario [3]. One important feature of event-triggered control is that it is more flexible and efficient than sampled-data control because it only executes a task in the event that is a pre-defined event-triggered condition that is violated [4]. This can significantly reduce the amount of information transmitted and control the updates. Therefore, an agreement between multi-agent systems with constrained sources is better suited for event-triggered control. In recent times, numerous significant outcomes pertaining to the agreement with event-triggered control system [5] have been acquired. The consent of first-order in multi-agent systems was presented by using both distributed and centralized event-triggered algorithms [6]. In generic linear dynamics and event-triggered control, the consensus problem was examined in [7]. In, [8] a distributed event-triggered following control problem with incomplete measurements and communication delays for leader-follower multi-agent systems was studied. Investigations on the containment control problem for first- and second-order multi-agent systems are conducted under the directed topology in [9] event-based broadcasting. The authors suggested distributed event-triggered control schemes for general linear multi-agent systems' finite-time consensus [10]. Examined the event-triggered consensus in [11] multi-agent system with external disturbance and estimated relative state measures. Scholars have done a great deal of research to try and determine the exact status of the vehicle. Since external signals are prone to mistakes and outages, estimation methods usually combine integral external measurements with data from other sensors [12]. The cooperative control issue of nonlinear leader-following multi-agent systems (MASs) has drawn a lot of interest and has been applied in a number of contexts, including formation control, unmanned aerial vehicles, sensor networks, smart grids, and sensor networks. In the leader-following MAS scenario, a distributed control protocol is used by all of the followers to track the leader's state trajectory using the available local neighborhood information [13]. It is harder to construct cooperative controllers for nonlinear systems—especially higher-order nonlinear systems—than for linear MASs in general. Researchers all throughout the world are also consistently becoming interested in related research projects [14]. A popular topic in the realm of mass analytics is finite-time consensus. A specified performance function based on neuro-adaptive cooperative tracking control for extremely non-linear mass amplitude systems [15]. The given necessary requirements for semi-stable finite-time consensus by combining the ideas of semi-stability and finite-time performance to nonlinear MASs [16]. A continuous state feedback-based distributed finite-time cooperative control mechanism is put forth [17]. Network systems first employed a time-triggered control method. On the other hand, this somewhat cautious approach uses a lot of network resources. In light of these facts, researchers suggested an event-triggered approach constructed on the system's current condition to reduce needless resource consumption. The fundamental concept of event-triggered control is to enhance efficiency by doing away with periodic or continuous sampling and only triggering events when an error in signal surpasses a reasonable upper bound. The event-triggered approaches in transportation are associated with measurement error. Typically, they gather reasonably accurate state information by using suitable sampling or estimate techniques, and they base their broadcast on whether the state inaccuracy surpasses the threshold [18]. A communication technique based on mistake correlation was initially proposed in 2010 [19]. After that, it was increasingly adopted and changed. Compared and thoroughly examined the methods that are now in use in recent years [20]. Cars can communicate their present condition to all other nearby cars at varied intervals thanks to the error-dependent approach. Consensus is a crucial concept in cooperative control, [21] which refers to the process by which a collection of agents [22], using distributed control protocols [23] based on local information [24], eventually agree on specific quantities of interest [25]. Numerous consensus algorithms have been created thus far to address a range of issues, [26] including time delays, [27] noisy measurement, [28] impulsive control, [29] and random switching typologies [30]. Cooperative control in multi-agent systems has garnered significant attention in current research because of its widespread adoption in various disciplines [31]. Smart grids and urban traffic signals are two examples. Consensus control attracted a lot of attention because it was a significant, disputed issue [32]. Research in the field of distributed artificial intelligence has primarily focused [33] on three areas: Distributed Problem Solving (DPS), [34] multi-agent System (MAS), [35] and parallel AI [36]. Parallel AI primarily refers to procedures that make classical AI approaches easier to employ [37] when applied to multiprocessor distributed hardware design [38] or cluster-based processing [39]. Distributed artificial intelligence (DAI) is a subfield of artificial intelligence which gained much importance because of its ability to solve complicated real-world problems.

Inspired by the discussion above, we described two contributions such that

(1) This paper presents event-triggered methods, both centralized and decentralized, designed for discrete-time multi-agent systems that include disturbances and delays in their input. These algorithms help in the containment control goal by directing the followers in the direction of convergence to the convex hull that the chosen leaders have established. Despite uncertainties in the system dynamics, the suggested algorithms provide a methodical way to manage input delays and disturbances, guaranteeing strong and efficient containment control.

(2) Important insights into the stability qualities of the system are revealed by an investigation of its behavior under the recommended procedures. This work thoroughly proves that the closed system does not show zeno behavior by using Lyapunov functions. This result emphasizes how well the suggested event-triggered confinement management mechanisms work overtime to keep the system stable. This work supports the efficiency of the suggested control protocols by using Lyapunov-based stability analysis to further our understanding of the behavior of the system when input delays and disturbances are present.

The structure of the remaining content is as follows:

1. In the section 1, we introduce the event-triggered containment with centralized and decentralized control laws established for discrete-time multi-agent system with input delay and disturbances, under some easy criteria composed as matrix inequalities, the consensus can be reached.
2. In the section 2 graph theory explained for the communication of agents.
3. In the section 3 provided a problem description, assumptions, some important remarks, and definition provided.
4. In the section 4 explained main results, also assumptions, event-triggered scheme, and important theorems.
5. In section 5 some numerical experiments are provided to show how effective the suggested approach.
6. In section 6 made conclusions.

**Notation.** Given that R represents the set of real numbers, $R^{n}$ and $R^{n_{1}\times n_{2}}$ indicate the n-dimensional real vector space and $n_{1}\times n_{2}$ real matrices, respectively. Here, $I_{n}$ stands for the n-dimensional identity matrix, and “T” signifies matrix transposition. The notation $\lambda_{max}(S)$ denotes the maximum eigenvalue of matrix *S*, where *S* is a real matrix. For a vector $z\in R^{n}$, its norm is described as $|z|=\sqrt{z^{T}z}$.

## 2 Preliminaries

### 2.1 Graph theory

In discrete-time multi-agent systems, communication topology is represented by communication graphs, G = (ν,ε). Where *ν* is the set of vertices that represent agents and *ε* is the set of edges that represent communication connections. An undirected network is made up of discrete-time multi-agents and a finite number of nodes, each of which represents a unique entity. Where ε⊆ν×ν and we define the adjacency matrix such that

$$a_{ij}=\left\{ \begin{array}{cc} 0, & \text{if }\varepsilon_{ij}\notin \varepsilon ,\\ 1, & \text{if }\varepsilon_{ij}\in \varepsilon , \end{array}\right.$$

Where A=${\left[a_{ij}\right]}^{N\times N}$ is the adjancey matrix. $\varepsilon_{ij}$ denotes the interaction between nodes i and j, where $a_{i0}$ and $a_{ii}=0$ represent follower communication with the leader. If follower receives information from the leader then $a_{ij}=1$ otherwise $a_{ij}=0$. The communication dynamics within the network can be expressed in this structure.

## 3 Problem description

In this paper, we take multi-agent systems which are controlled through a well-designed control protocol. Consider that there are K follower agents and l-K leader agents. The network topology among K agents which are controlled by a directed graph G(ν,ϵ). Let $v_{i}\in R$ be the position of i-agents. Now the dynamics of follower agents are such that

(1) $$v_{i}(\overset{˜}{m}+1)=v_{i}(\overset{˜}{m})+(u_{i})(\overset{˜}{m}-\overset{˜}{n})+△u_{i}(\overset{˜}{m})\;\forall i\in M_{F}.$$

Where $u_{i}(\overset{˜}{m}-\overset{˜}{n})$ is control input with communication lag and $△u_{i}(\overset{˜}{m})$ is disturbance in control input. Now the dynamics of leader agent l.

(2) $$v_{i}(\overset{˜}{m}+1)=v_{i}(\overset{˜}{m})\;\forall i\in M_{L}.$$

Now, we assume that our leader agent is fixed.

Assumption 3.1 It is considered that the communication network $\left(G_{F}\right)$ is undirected. We assumed there exists a leader agent that is assigned and able to provide a path to each follower agent while taking into consideration input delays and disruptions in control inputs.

Remark 3.1 This assumption is crucial to networked control systems because it guarantees that routes of communication will be established between the leader and followers agents despite delays and disruptions. Assume that their communication network is undirected and that the leader agent can still reach every follower agent in spite of these obstacles and assumption makes it easier to create strong control schemes for the networked system.

Assumption 3.2 Matrix $L_{1}$ is confirmed as positive definite, while the product $-L_{1}^{-1}L_{2}$ is recognized as a stochastic matrix.

Definition 3.1 This control technique aims to ensure that every follower converges upon $co(V_{L})$, the convex hull of the leader's agents such that

$$\lim_{(\overset{˜}{m}-\overset{˜}{n})\to \infty }\left\|v_{i}(\overset{˜}{m}-\overset{˜}{n})-co(M_{L})\right\|=0,\;\forall i\in M_{F}.$$

For the system, we define confinement control as existing if and only if the follower's agents converge to $co(M_{L})$.

Remark 3.2 The Laplacian matrix L has a distinct if and only if the graph G has zero eigenvalue a linked sub-graph called a spanning tree that has all of its vertices connected by the least amount of edges. This characteristic denotes the fundamental interaction of the graph. L has a linked component in the graph since it has at least one zero eigenvalue and an eigenvector of all ones. This condition supports stability in dynamic systems illustrated by the graph, as do all non-zero eigenvalues with positive real components.

Definition 3.2 A stochastic matrix is non-negative and has row sums of +1, whereas matrix A becomes non-negative if all of its members are non-negative. Considering a limited collection of vectors $v_{1},v_{2},...,v_{n}\in R^{p}$, their convex hull is defined as $co\{v_{1},v_{2},...,v_{n}\}=\{\sum_{i=1}^{n}\beta_{i}v_{i}|v_{i}\in M,\beta_{i}\geq 0,\sum_{i=1}^{n}\beta_{i}=1\}$. For $v\in R^{p}$ and $U\subset R^{p}$, ‖v−U‖ denotes the distance between *x* and *U*, represented as $\inf_{u\in U}{\left\|v-u\right\|}_{2}$. This notation defines the minimum Euclidean distance between *v* and any point in *U*.

## 4 Main results

Two different event-triggered control protocols have been designed which are specially designed to deal with the challenges caused by input delays and disturbances in the containment control problem. Because without ignoring these problems we can design a reliable and advanced controller. In the centralized technique, a robust protocol is designed so that follower agents are subject to interruptions and input lags/delays and also adjust their control inputs only when predefined trigger points are met under predefined conditions. To preserve robustness, the decentralized protocol allows followers to effectively handle disruptions and input delays while maintaining stability throughout the time between trigger instances. To improve system performance and dependability, these protocols provide a systematic structure for handling the complexity involved in confinement control.

First of all, we define a centralized control protocol with input delays and disturbances such that

(3) $$z_{i}(\overset{˜}{m}-\overset{˜}{n})=-\gamma \sum_{j=1}^{n}a_{ij}[(v_{i}{\left(\overset{˜}{m}-\overset{˜}{n}\right)}_{r}-v_{j}{\left(\overset{˜}{m}-\overset{˜}{n}\right)}_{r}]+\Delta z_{i}(\overset{˜}{m}),\;\forall \overset{˜}{m}\in [{\overset{˜}{m}}_{r},{\overset{˜}{m}}_{r+1})$$

Similarly, we designed a most reliable decentralized control protocol with input delay and disturbance such that

(4) $$z_{i}(\overset{˜}{m}-\overset{˜}{n})=-\gamma \sum_{j=1}^{n}a_{ij}\left[v_{i}{\left(\overset{˜}{m}-\overset{˜}{n}\right)}_{r}^{i}-v_{j}{\left(\overset{˜}{m}-\overset{˜}{n}\right)}_{r^{⁎}}^{j})+\Delta z_{i}(\overset{˜}{m})\right],$$

Where *γ* is a feedback gain and $\Delta z_{i}(\overset{˜}{m})$ is the disturbance.

Assumption 4.1 Assume in the system that their leader agent remains static. It emphasizes the centralized identity of information transmission because followers can only receive the leader's information if they are nearby agents. The dynamics of decision-making among network followers are shaped by this process based on physical connection.

Now we define the state vector for followers agents such that

$$v_{F}(\overset{˜}{m}-\overset{˜}{n})={\left(v_{1}(\overset{˜}{m}-\overset{˜}{n}),v_{2}(\overset{˜}{m}-\overset{˜}{n}),...,v_{m}(\overset{˜}{m}-\overset{˜}{n})\right)}^{T}$$

Similarly, we define the state vector for leader agent by the use of Eq(2) which is nearby such that

$$v_{R}(\overset{˜}{m}-\overset{˜}{n})={\left(v_{m+1}(\overset{˜}{m}-\overset{˜}{n}),v_{m+2}(\overset{˜}{m}-\overset{˜}{n}),...,v_{n}(\overset{˜}{m}-\overset{˜}{n})\right)}^{T}$$

Using eq(3) in eq(1) we get

(5) $$v_{F}(\overset{˜}{m}+1)=v_{F}(\overset{˜}{m})-\gamma L_{1}v_{F}(\overset{˜}{m}-\overset{˜}{n})-\gamma L_{2}v_{R}(\overset{˜}{m}-\overset{˜}{n})+2\Delta z_{i}(\overset{˜}{m})$$

Now we define the tracking error such that

$$v_{F}(\overset{˜}{m}-\overset{˜}{n})=v_{F}(\overset{˜}{m}-\overset{˜}{n})+L_{1}^{-1}L_{2}v_{R}(\overset{˜}{m}-\overset{˜}{n})$$

and define measurement error such that

$$e_{F}(\overset{˜}{m}-\overset{˜}{n})={\left(e_{1}(\overset{˜}{m}-\overset{˜}{n}),e_{2}(\overset{˜}{m}-\overset{˜}{n}),...,e_{m}(\overset{˜}{m}-\overset{˜}{n})\right)}^{T}$$

and we take

$$e_{i}(\overset{˜}{m}-\overset{˜}{n})=v_{i}{\left(\overset{˜}{m}-\overset{˜}{n}\right)}_{r}-v_{i}(\overset{˜}{m}-\overset{˜}{n})\;i\in M_{F}$$

By using tracking error and measurement error in eq(5) and, we get

(6) $$v_{F}(\overset{˜}{m}+1)=(I-\gamma L_{1})v_{F}(\overset{˜}{m}-\overset{˜}{n})-\gamma L_{1}(e_{F}(\overset{˜}{m}-\overset{˜}{n})+2\Delta z_{i}(\overset{˜}{m})$$

Similarly for decentralized technique by using eq(4) in eq(1). We conclude that

(7) $$v_{F}(\overset{˜}{m}+1)=v_{i}(\overset{˜}{m})-\gamma L_{1}v_{F}(\overset{˜}{m}-\overset{˜}{n})-\gamma L_{2}v_{R}-\gamma L_{1}e(\overset{˜}{m}-\overset{˜}{n})+2\Delta z_{i}(\overset{˜}{m})$$

We take $v_{F}(\overset{˜}{m})-\overset{˜}{n})=v_{F}(\overset{˜}{m}+L_{1}^{-1}L_{2}v_{R})$ and using in eq(7), we get

(8) $$v_{F}(\overset{˜}{m}+1)=(I-\gamma L_{1})v_{F}(\overset{˜}{m}-\overset{˜}{n})-\gamma L_{1}e(\overset{˜}{m}-\overset{˜}{n})+2\Delta z_{i}(\overset{˜}{m})$$

### 4.1 Event triggered scheme

We design the event-triggered condition such that

(9) $$\left\|e_{F}(\overset{˜}{m}-\overset{˜}{n})\|\geq \psi \|L_{1}v_{F}((\overset{˜}{m}-\overset{˜}{n})\right\|$$

If the condition meets then $\left\|e_{F}(\overset{˜}{m}-\overset{˜}{n})\|\geq \psi \|L_{1}v_{F}((\overset{˜}{m}-\overset{˜}{n})\right\|$. When followers update their control inputs, it prompts a reset of measurement errors to zero. This change is crucial for sustaining the accuracy and reliability of subsequent control actions. Conversely, without these updates, followers' control inputs remain unchanged. This mechanism is essential for maintaining the stability and adaptability of the control system amidst changing environmental conditions. Measurement error satisfies if $\left\|e_{F}(\overset{˜}{m}-\overset{˜}{n})\|\leq \psi \|L_{1}v_{F}((\overset{˜}{m}-\overset{˜}{n})\right\|$. It also preserves that this condition eq(7) for centralized control input.

Similarly, we design a event-triggered condition for the decentralized technique such that

(10) $$\left\|e_{i}(\overset{˜}{m}-\overset{˜}{n})\|\geq \psi \|v_{F}^{i}(\overset{˜}{m}-\overset{˜}{n})\right\|$$

Where *ψ* is a positive constant.

Assumption 4.2 We assume that within a graph without direction G, a minimum of one leader agent is present capable of establishing a direct or indirect path to each follower agent

Theorem 4.1 *Assume that our* Assumption 4.2 *satisfies only for* $0<\gamma <2\frac{1}{\left\|L_{1}\right\|},0<\psi <\frac{(1+\gamma \|L_{1}\|)+\sqrt{1+4\gamma \|L_{1}\|}}{\gamma \|L_{1}\|}$ *. Using the centralized control input eq* (3) *and event-triggered scheme eq* (9) *, satisfy and achieve containment control for the system eq* (1) *.*

Proof We choose a Lyapunov function

$$P(\overset{˜}{m})=v_{F}^{T}(\overset{˜}{m})L_{1}v_{F}(\overset{˜}{m})$$

Determining the $P(\overset{˜}{m})$ difference throughout the follower dynamics of eq(6).

$$\nabla P=v_{F}^{T}(\overset{˜}{m}+1)L_{1}v_{F}(\overset{˜}{m}+1)-v_{F}^{T}L_{1}v_{F}(\overset{˜}{m})+2\Delta z_{i}(\overset{˜}{m})$$

$$=-2\gamma v_{F}^{T}(\overset{˜}{m}-\overset{˜}{n})L_{1}^{2}v_{F}(\overset{˜}{m}-\overset{˜}{n})-2\gamma v_{F}^{T}(\overset{˜}{m}-\overset{˜}{n})L_{1}^{2}e_{F}(\overset{˜}{m}-\overset{˜}{n})\;+\gamma^{2}v_{F}^{T}(\overset{˜}{m}-\overset{˜}{n})L_{1}^{3}v_{F}(\overset{˜}{m}-\overset{˜}{n})+2\gamma^{2}v_{F}^{T}(\overset{˜}{m}-\overset{˜}{n})L_{1}^{3}e_{F}(\overset{˜}{m}-\overset{˜}{n})\;+\gamma^{2}e_{F}^{T}(\overset{˜}{m}-\overset{˜}{n})L_{1}^{3}e_{F}(\overset{˜}{m}-\overset{˜}{n})+2\Delta z_{i}(\overset{˜}{m})$$

$$\leq -\gamma {\left\|L_{1}v_{F}(\overset{˜}{m}-\overset{˜}{n})\right\|}^{2}+2\gamma \|L_{1}\|\|L_{1}v_{F}(\overset{˜}{m}-\overset{˜}{n})\|\|e_{F}(\overset{˜}{m}-\overset{˜}{n})\;+\gamma^{2}\|L_{1}\|{\left\|L_{1}V_{F}(\overset{˜}{m}-\overset{˜}{n})\right\|}^{2}+2\gamma^{2}{\left\|L_{1}\right\|}^{2}\|L_{1}V_{F}(\overset{˜}{m}-\overset{˜}{n})\|\times \|e_{F}(\overset{˜}{m}-\overset{˜}{n})\|\;+\gamma^{2}{\left\|L_{1}\right\|}^{3}{\left\|e_{F}(\overset{˜}{m}-\overset{˜}{n})\right\|}^{2}+2\Delta z_{i}(\overset{˜}{m})$$

Now by using the event-triggered condition such that

(11) $$\left\|e_{F}(\overset{˜}{m}-\overset{˜}{n})\|\geq \psi \|L_{1}v_{F}((\overset{˜}{m}-\overset{˜}{n})\right\|$$

By applying this condition eq(11), we satisfy that

$$\nabla P\leq \gamma \zeta (\psi ){\left\|L_{1}v_{F}(\overset{˜}{m}-\overset{˜}{n})\right\|}^{2}$$

We denote here that $\zeta (\psi )=\gamma {\left\|L_{1}^{3}\right\|}^{3}+2\|L_{1}\|(1+\gamma \|L_{1})\|\psi +\gamma \|L_{1}\|-2$. It is obvious that there are distinct real roots for the equation ζ(ψ)=0. Thus $0<\gamma \frac{2}{\left\|L_{1}\right\|}$, $\psi_{1}$, $\psi_{2}$ can be derived.

By solving it we get

$$\psi_{1}=\frac{(1+\gamma \|L_{1})+\sqrt{1+4\gamma \|L_{1}\|}}{\gamma \|L_{1}\|}$$

and

$$\psi_{2}=\frac{(1+\gamma \|L_{1})+\sqrt{1+4\gamma \|L_{1}\|}}{\gamma \|L_{1}\|}$$

We conclude that ζ(ψ)<0 when $\psi <\psi_{1}$.

As a result, we get

$$\nabla P\leq \gamma \zeta (\psi ){\left\|L_{1}v_{F}(\overset{˜}{m}-\overset{˜}{n})\right\|}^{2}<0$$

Using the assumption (3.2) and using the definition (3.1), we conclude that and satisfy that it converges.

$$\lim_{(\overset{˜}{m}-\overset{˜}{n})\to \infty }\left\|v_{i}(\overset{˜}{m}-\overset{˜}{n})-co(M_{L})\right\|=0,\;\forall i\in M_{F}.$$

 □

Remark 4.2 Also, preserve that the gap between event instants $\overset{˜}{m}$ and ${\overset{˜}{m}}_{r+1}$ is guaranteed to be at least 1 if condition (9) is satisfied. A non-accumulative cycle of events is guaranteed by the closed-loop system with the avoidance of Zeno behavior.

Theorem 4.3 *Take the assumption* (4.2) *satisfy that 0<* $\gamma <2\frac{1}{\left\|L_{1}\right\|},0<\psi <\frac{(1+\gamma \|L_{1})+\sqrt{1+4\gamma \|L_{1}\|}}{\gamma \|L_{1}\|}$ *. Now using the decentralized control input eq* (4) *using the condition eq* (10) *obtained the system eq* (1) *.*

Proof Again choose the same Lyapunov function

$$P(\overset{˜}{m})=v_{F}^{T}(\overset{˜}{m})L_{1}v_{F}(\overset{˜}{m})$$

Similarly taking the difference of the dynamics and by using eq(8)

$$\nabla P=v_{F}^{T}(\overset{˜}{m}+1)L_{1}v_{F}(\overset{˜}{m}+1)-v_{F}^{T}L_{1}v_{F}(\overset{˜}{m})+2\Delta z_{i}(\overset{˜}{m})$$

$$\leq -\gamma {\left\|L_{1}v_{F}(\overset{˜}{m}-\overset{˜}{n})\right\|}^{2}+2\gamma \|L_{1}\|\|L_{1}v_{F}(\overset{˜}{m}-\overset{˜}{n})\|\|e_{F}(\overset{˜}{m}-\overset{˜}{n})\;+\gamma^{2}\|L_{1}\|{\left\|L_{1}V_{F}(\overset{˜}{m}-\overset{˜}{n})\right\|}^{2}+2\gamma^{2}{\left\|L_{1}\right\|}^{2}\|L_{1}V_{F}(\overset{˜}{m}-\overset{˜}{n})\|\times \|e_{F}(\overset{˜}{m}-\overset{˜}{n})\|\;+\gamma^{2}{\left\|L_{1}\right\|}^{3}{\left\|e_{F}(\overset{˜}{m}-\overset{˜}{n})\right\|}^{2}+2\Delta z_{i}(\overset{˜}{m})$$

By satisfying the event triggered condition eq(10).

Using a method similar to Theorem (4.1), the other part of this proof can be completed. □

Remark 4.4 The decentralized event triggered condition eq(10) updates only the nearby agent information of each follower i and control input minimizes the control updates. Also, note that the closed-loop system does not exhibit the zeno behavior.

## 5 Numerical experiments

In the numerical simulations, two examples are given to clarify the mathematical outcomes.

**Figure 1:**
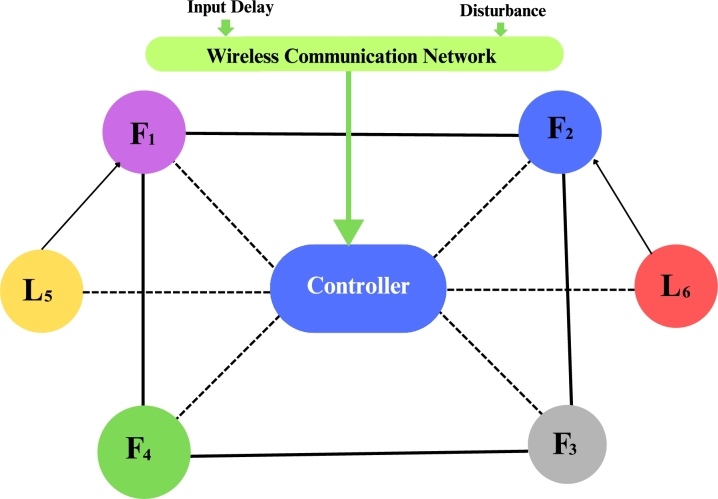
Network topology of agents.

Consider that there are four followers multi agents such that $F_{1}$, $F_{2}$, $F_{3}$, and $F_{4}$ and two leader agents denoted by $L_{5}$ and $L_{6}$. Network communication topology has shown the influence of input delay and disturbances in Fig. 1 and we take the value of γ=0.8, ψ=0.02, $\Delta z_{i}(\overset{˜}{m})=3\sin(0.8)$ and input delay $\overset{˜}{n}=0.5$.

**Figure 2:**
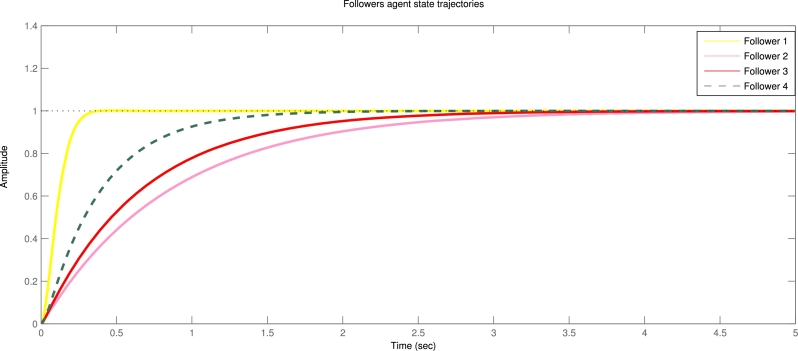
State trajectories of four followers with centralized event triggered control input.

**Figure 3:**
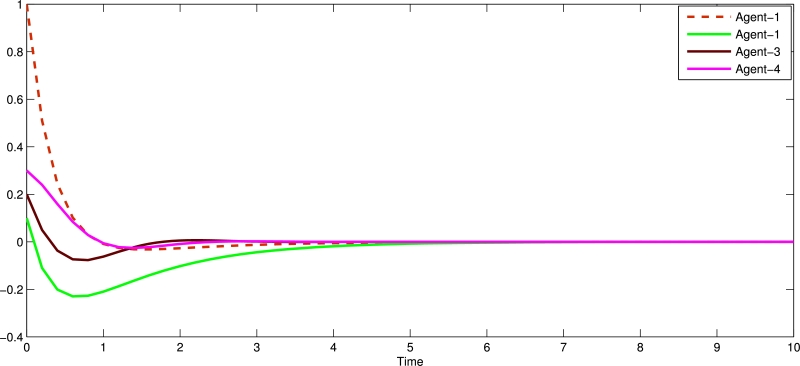
Shows state trajectories without of centralized event triggered control system for four followers agents.

Example 5.1 We take the centralized control strategy with input delay and disturbance. Fig. 2 indicates state trajectories of four followers agents and uses centralized control input with input delay and disturbance (3) with event-triggered condition (9). Fig. 3 shows the state trajectories without of centralized event triggered control system for four followers agents. Assume that leader agents are stationary. Also note that we measured time per seconds for all figures. As in Fig. 2 measured time per seconds. Similarly all figures time measures per seconds.

Define the Laplacian matrix such that

$$L_{1}=\left(\begin{array}{cccc} 4 & -3 & 0.1 & -3\\ -3 & 4 & -3 & 0.1\\ 0.1 & -3 & 4 & -3\\ -3 & 0.1 & -3 & 4 \end{array}\right),\;L_{2}=\left(\begin{array}{cccc} -3 & -0.1 & 0 & 0\\ 0 & 0 & -1 & 3\\ -4 & 0 & 0 & 0\\ 5 & 1 & 0.1 & 4 \end{array}\right)$$

**Figure 4:**
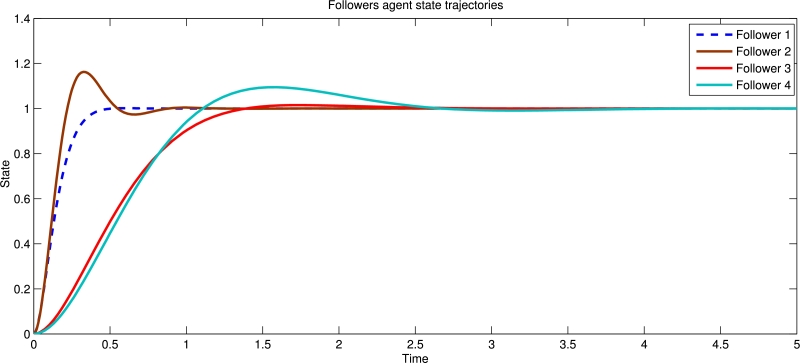
State trajectories of four followers with decentralized event triggered control input.

**Figure 5:**
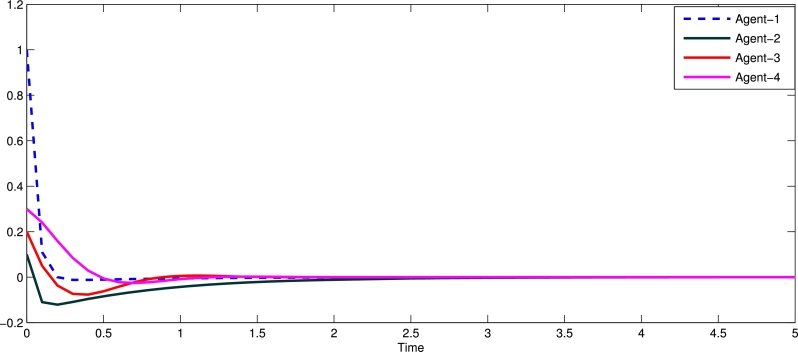
Shows state trajectories without of decentralized event triggered control system for four followers agents.

Example 5.2 Similarly, we take decentralized control input with input delay and disturbance (4) and by using event-triggered condition (10). Fig. 4 shows the state trajectories with decentralized control input and also proves that containment control obtained. Fig. 5 shows the state trajectories without of decentralized event triggered control system for four follower agents.

## 6 Conclusion

The article investigates discrete-time multi-agent systems' event-triggered confinement management while taking disturbance and delay into account. First, an event-triggered control protocol with input delay and disturbance and a corresponding triggering condition is presented. The centralization of the control strategy stems from the requirement for global information for every agent, which takes disturbance and delay into consideration. The analysis shows that the suggested technique successfully accomplishes containment control when followers have an undirected network topology and each follower has at least one leader connected to it. It's a proven result that zeno behavior is not exhibited by the closed-loop system. Next, a decentralized event-triggered method that takes input delay and disturbances into account is introduced, requiring just the nearby agents' states for each follower. The research also offers enough information to guarantee efficient containment management. Future research could extend these control strategies to directed networks and more complex disturbance models. An adaptive event-triggering mechanisms that dynamically adjust to the system's state could improve containment control efficiency and robustness.

## CRediT authorship contribution statement

**Hanen Louati:** Project administration. **Azmat Ullah Khan Niazi:** Supervision, Conceptualization. **Mhassen. E.E. Dalam:** Formal analysis. **Waqar Ul Hassan:** Writing – original draft, Software. **Khawer Hameed Khan:** Writing – review & editing, Data curation. **Mohammed Alhagyan:** Resources.

## Declaration of Competing Interest

The authors declare that they have no known competing financial interests or personal relationships that could have appeared to influence the work reported in this paper.

## Data Availability

The dataset supporting the conclusions of this article is included within the article.
